# The Compartmentalisation of Phosphorylated Free Oligosaccharides in Cells from a CDG Ig Patient Reveals a Novel ER-to-Cytosol Translocation Process

**DOI:** 10.1371/journal.pone.0011675

**Published:** 2010-07-20

**Authors:** Delphine Peric, Christelle Durrant-Arico, Christophe Delenda, Thierry Dupré, Pascale De Lonlay, Hélène Ogier de Baulny, Cécile Pelatan, Brigitte Bader-Meunier, Olivier Danos, Isabelle Chantret, Stuart E. H. Moore

**Affiliations:** 1 INSERM U773 CRB3, Paris, France; 2 Université Denis Diderot, Paris 7, Paris, France; 3 Université René Descartes, Paris 5, Paris, France; 4 Généthon: Evry, France; 5 AP-HP, Hôpital Bichat-Claude Bernard, Biochimie Métabolique et Cellulaire, Paris, France; 6 Département de Pédiatrie, Hôpital Necker-Enfants Malades, Paris, France; 7 Neurologie et maladies métaboliques, Hôpital Robert Debré, APHP, Paris, France; 8 Centre Hospitalier, Service de Pédiatrie, Le Mans, France; 9 Service d'Hématologie Biologique, Hôpital de Bicêtre, APHP, Paris, France; 10 INSERM U781, Hôpital Necker-Enfants Malades, Paris, France; Massachusetts Institute of Technology, United States of America

## Abstract

**Background:**

Biosynthesis of the dolichol linked oligosaccharide (DLO) required for protein N-glycosylation starts on the cytoplasmic face of the ER to give Man_5_GlcNAc_2_-PP-dolichol, which then flips into the ER for further glycosylation yielding mature DLO (Glc_3_Man_9_GlcNAc_2_-PP-dolichol). After transfer of Glc_3_Man_9_GlcNAc_2_ onto protein, dolichol-PP is recycled to dolichol-P and reused for DLO biosynthesis. Because *de novo* dolichol synthesis is slow, dolichol recycling is rate limiting for protein glycosylation. Immature DLO intermediates may also be recycled by pyrophosphatase-mediated cleavage to yield dolichol-P and phosphorylated oligosaccharides (fOSGN2-P). Here, we examine fOSGN2-P generation in cells from patients with type I Congenital Disorders of Glycosylation (CDG I) in which defects in the dolichol cycle cause accumulation of immature DLO intermediates and protein hypoglycosylation.

**Methods and Principal Findings:**

In EBV-transformed lymphoblastoid cells from CDG I patients and normal subjects a correlation exists between the quantities of metabolically radiolabeled fOSGN2-P and truncated DLO intermediates only when these two classes of compounds possess 7 or less hexose residues. Larger fOSGN2-P were difficult to detect despite an abundance of more fully mannosylated and glucosylated DLO. When CDG Ig cells, which accumulate Man_7_GlcNAc_2_-PP-dolichol, are permeabilised so that vesicular transport and protein synthesis are abolished, the DLO pool required for Man_7_GlcNAc_2_-P generation could be depleted by adding exogenous glycosylation acceptor peptide. Under conditions where a glycotripeptide and neutral free oligosaccharides remain predominantly in the lumen of the ER, Man_7_GlcNAc_2_-P appears in the cytosol without detectable generation of ER luminal Man_7_GlcNAc_2_-P.

**Conclusions and Significance:**

The DLO pools required for N-glycosylation and fOSGN2-P generation are functionally linked and this substantiates the hypothesis that pyrophosphatase-mediated cleavage of DLO intermediates yields recyclable dolichol-P. The kinetics of cytosolic fOSGN2-P generation from a luminally-generated DLO intermediate demonstrate the presence of a previously undetected ER-to-cytosol translocation process for either fOSGN2-P or DLO.

## Introduction

**Figure 1:**
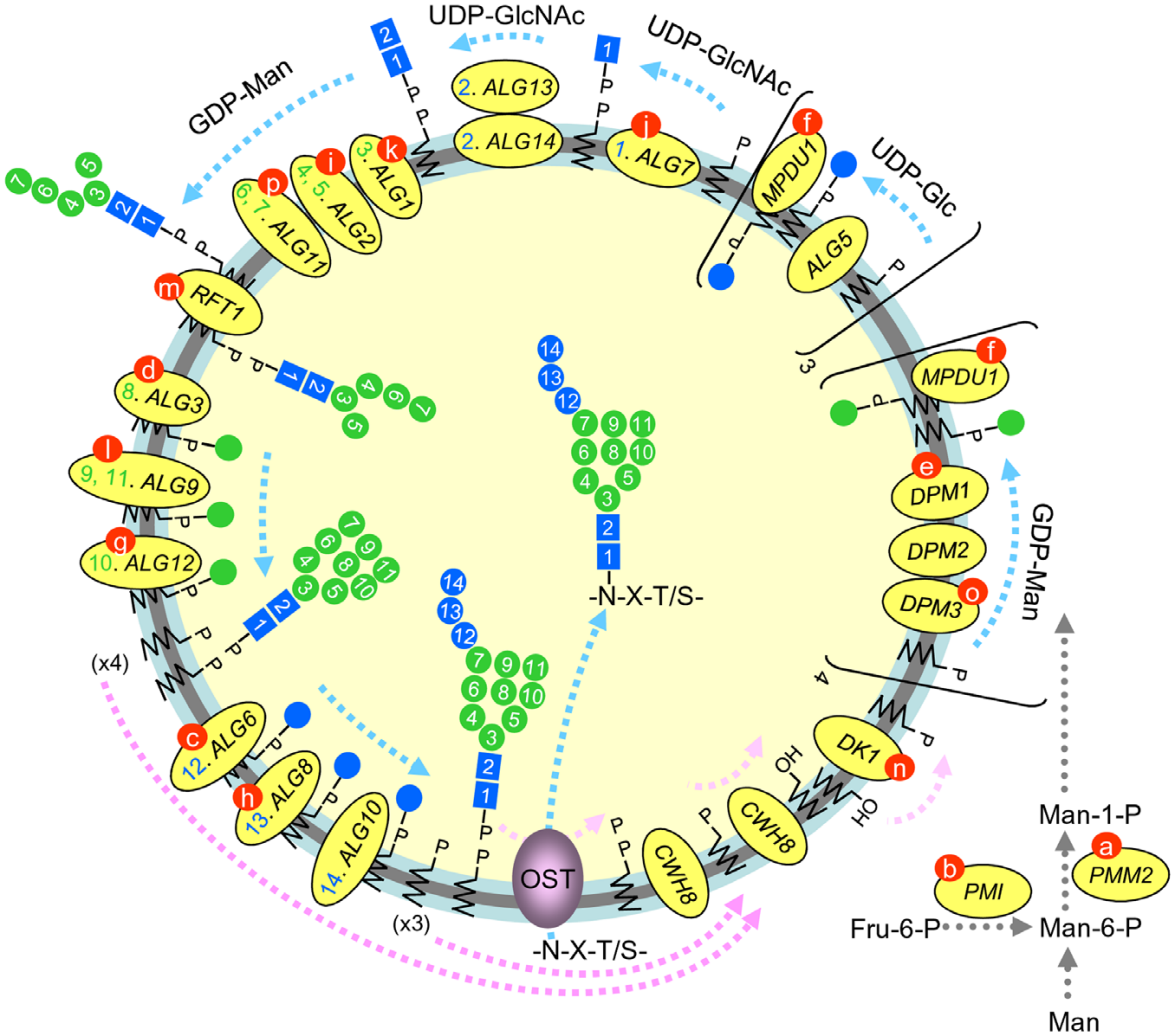
The dolichol cycle and protein N-glycosylation. The dolichol cycle consists of a series of reactions (heavy dashed blue lines) involved in the construction of the oligosaccharide precursor (Glc_3_Man_9_GlcNAc_2_) on a dolichol carrier. The cycle is completed by reactions (heavy dashed pink lines) involved in the recycling of dolichol-phosphate (dol-P). Mature lipid linked oligosaccharide (DLO) is generated by the transfer of two residues of N-acetylglucosamine (blue squares), 9 residues of mannose (green circles) and 3 residues of glucose (blue circles) onto the lipid carrier dolichol-P (zig zag line). The monosaccharides are added sequentially, in the order indicated by their numbers, by glycosyltransferases whose gene names are shown in yellow ovals and whose order of action is also indicated. The first seven sugars are added by cytoplasmically orientated UDP-GlcNAc- and GDP-Man-requiring glycosyltransferases. The growing DLO is then flipped into the lumen of the ER by a process thought to involve the RFT1 gene product. Subsequently dolichol-P-Man (DPM)- and dolichol-P-Glc (DPG)-requiring glycosyltransferases complete DLO biosynthesis. The addition of the last glucose residue to the growing DLO allows efficient oligosaccharyltransferase (OST)-mediated transfer of the oligosaccharide from lipid onto nascent polypeptides (-N-X-T/S-) in the ER. As indicated by the heavy dashed pink lines, a series of reactions carried out by gene products indicated in the yellow ovals, ensure that the lumenally orientated dol-P and dol-PP molecules that are generated during the construction of mature DLO are reorientated towards the cytosolic face of the ER. The different type I congenital disorders of glycosylation subtypes (CDG Ia-p, indicated by letters in red circles on the gene name) are caused by mutations in genes encoding enzymes involved in either the construction of mature DLO or dolichol recycling.

The majority of secretory and cell surface glycoproteins are N-glycosylated by the co-, or post-translational addition of the oligosaccharide, Glc_3_Man_9_GlcNAc_2_, that is transferred from the mature dolichol-linked oligosaccharide (DLO), Glc_3_Man_9_GlcNAc_2_-PP-dolichol, onto nascent polypeptides in the lumen of the endoplasmic reticulum (ER) by oligosaccharyltransferase (OST, see Fig. 1). Luminally orientated dolichol-PP, the by product of OST-mediated protein glycosylation (Fig. 1), is recycled to yield dolichol-P oriented on the cytoplasmic face of the ER [1]. Dolichol-P is consumed during 3 reactions occurring on the cytoplasmic face of the ER membrane (Fig. 1) that lead to the generation of dolichol-PP-GlcNAc, dolichol-P-Man (DPM) and dolichol-P-Glc (DPG) [2], [3]. The former molecule is now elongated to yield Man_5_GlcNAc_2_-PP-dolichol by cytoplasmically orientated, UDP-GlcNAc-, and GDP-Man-requiring, glycosyltransferases [4], [5]. After flipping into the lumen of the ER [6], [7], [8], the growing DLO is completed by DPM- and DPG-requiring glycosyltransferases, whose active sites are thought to face the lumen of the ER [9], [10], to yield the mature DLO. The ensemble of these reactions constitutes the dolichol cycle and its interruption leads to hypoglycosylation of glycoproteins in yeast [11] and mammalian cells [12]. In the human population mutations in genes involved in the dolichol cycle (Fig. 1) lead to rare inherited diseases called type I congenital disorders of glycosylation (CDG I). Of particular interest for the study of these diseases is the fate of accumulating immature DLO intermediates that could potentially tie up substantial quantities of limiting dolichol-P [2]. In fact, two processes leading to destruction of DLO, and thereby potentially promoting dolichol recycling, have been described. The first process [13], [14], [15] leads to the release of neutral free oligosaccharides (fOS) bearing two N-acetylyclucosamine (GlcNAc) residues at their reducing termini (fOSGN2). It is thought that when acceptor polypeptides are limiting, OST can transfer the Glc_3_Man_9_GlcNAc_2_ moiety of mature DLO onto water molecules to generate fOSGN2 [13], [14]. OST is preferentially active towards fully glucosylated and mannosylated DLO, leading to the production of the fOSGN2 Glc_3_Man_9_GlcNAc_2_ [13], [15]. During the second process [16], [17], [18] a pyrophosphatase activity [19] is thought to generate phosphorylated oligosaccharides (fOSGN2-P) and dolichol-P from non-glucosylated DLO intermediates [20], [21].

In the present study we investigated fOSGN2-P generation in EBV-transformed lymphoblasts derived from several CDG I patients as well as different murine lymphoblasts. In all cell lines, the fOSGN2-P pool comprised structures containing mainly 7 or less hexose residues (Hex_1-7_GlcNAc_2_-P) despite the relative abundance of more fully mannosylated and glucosylated DLO intermediates. An *in vitro* assay revealed that DLO pools that give rise to either fOSGN2-P or N-glycans are functionally linked. Furthermore, DLO intermediates synthesized in the lumen of the ER can give rise to cytosolic fOSGN2-P without detectable generation of ER-situated fOSGN2-P intermediates.

## Materials and Methods

### Ethics statement

Experiments on human cell lines were conducted in accordance with local ethics comittees and the Comités de Protection des Personnes (CPP, http://www.recherche-biomedicale.sante.gouv.fr/index.htm). After obtention of signed written parental consent forms, lymphoblasts derived from patients with unknown disease were immortalised with the Epstein Barr virus (EBV) as previously described [22].

### Reagents

D-mannitol was from Fluka (St Quentin Fallavier, France). [2-^3^H (N)]mannose (24.7 Ci/mmol), D-[6-^3^H (N)]glucosamine (25.9 Ci/mmol) and En^3^hance spray were from PerkinElmer Life Sciences (Zaventem, BE). TLC plates were obtained from MERCK (Darmstadt, DE). AG 50-X2 (H^+^ form) and AG 1-X2 (acetate form) came from Biorad SA, (Marnes la Coquette, FR). Streptolysin O (SLO) was a generous gift from Sucharit Bhakdi (Institute of Medical Microbiology and Hygiene, Mainz, DE). Fucose, endo-β-N-acetylglucosaminidase H from *Streptomyces plicatus* (endoH), protease and alkaline phophatase were purchased from SIGMA–Aldrich SARL (St Quentin Fallavier, FR). Castanospermine, kifunensin and swainsonine were from Toronto Research Chemicals Inc. (Toronto, CA). The tripeptide, Ac-NYT-NH_2_, was synthesised and purified [23] by Neosystem, Strasbourg, FR.

### Cell culture and metabolic radiolabelling procedures

The parental BW5147.3 and the Thy^-1^ negative, DPM1-deficient, mouse lymphoma cell lines [24], [25] (ATCC, Rockville, MD) and EBV-transformed cell lines were cultivated in RPMI 1640 Glutamax™ medium containing 10% fetal calf serum and 1% penicillin/streptomycin at 37°C under an atmosphere containing 5% CO_2_. The human cells used in this report are derived from normal subjects or patients diagnosed with CDG Ia (PMM2 mutations: p.Ile132Thr/p.Arg123Gln [26]), CDG Ie [27], CDG Ig [28] and CDG Ih [22]. Cells were maintained at densities of between 2×10^5^ and 2×10^6^ cells/ml. For metabolic radiolabelling, 8×10^7^ cells were harvested and then rinsed with glucose-free RPMI 1640 medium containing 0.5 mM glucose, 1.0 mM fucose and 2% dialysed fetal calf serum. Subsequently, cells were incubated in 1 ml of the same medium containing 20–100 µCi [2-^3^H (N)] mannose for 30 min at 37°C under an atmosphere containing 5% CO_2_. Where indicated, cells were preincubated in radiolabelling media containing 2 mM castanospermine (CST), 100 µM swainsonine (SW) or 100 µM kifunensin (KIF) for 45 min prior to addition of the radioactive sugars.

### Transduction of lymphoblasts with HIV-1-derived lentiviral vectors

The transfer vector encoding wild type hALG12 and enhanced green fluorescent protein (eGFP) has already been described [28]. Briefly, it consists of a bicistronic expression vector (pSIN.PW.hALG12.IRES2.eGFP) from which the mRNA is driven by the phosphoglycerate kinase promoter and into which the eGFP protein is translated via the IRES element from encephalopathy myocardiditis virus (EMCV). Transfer vector particles were produced by cotransfection of this transfer vector into human kidney 293T cells along with the packaging (Gag-Pol and Rev), and envelope (glycoprotein from the vesicular stomatitis virus (VSV/G)) constructs. Patient lymphoblast cells were transduced at different multiplicities of infection (MOIs) and eGFP-positive cells were sorted by FACS.

### Cell permeabilisation

After radiolabelling, cells were permeabilised using a modification of a previously described method [29], [30]. Briefly, cells were washed with ice cold phosphate buffered saline (PBS) and then with permeabilisation buffer (PB): 20 mM HEPES-KOH, pH 7.3, containing 250 mM mannitol and 1 mM CaCl_2_. The cells were then incubated for 1 h at 4°C in PB containing 2 µg/ml streptolysin O (SLO). Permeabilised cells containing membrane bound compartments (MBC) were then separated from the SLO perfusate containing cytoplasmic components (Cyt) by centrifugation at 130 gAv for 5 min at 4°C.

### *In vitro* assay for fOSGN2-P generation

SLO-permeabilised cells were incubated in an intracellular buffer (IB) as previously described [22], [31]. After washing with ice cold PBS, radiolabelled cells were washed into IB: 5 mM HEPES-KOH, pH 7.3, containing 130 mM K^+^/glutamate, 10 mM NaCl, 2 mM EGTA, 1 mM CaCl_2_ and 2 mM MgCl_2_. Subsequently, the cells were incubated on ice for 30 min in IB, containing 2 µg/ml SLO, and then washed twice with IB to remove excess SLO. Finally, the cells were incubated for 5 min in IB prewarmed to 37°C before a final wash into ice cold IB. Aliquots of the permeabilised cells were incubated with IB containing the various additions indicated in the figure legends in a reaction volume of 50 µL for different times at 37°C. Reactions were stopped by the addition of 450 µL ice cold IB. MBC and Cyt fractions were obtained as described above.

### Recovery of fOS, fOSGN2-P, glycoproteins and DLO from radiolabeled cells

These methods have all been adapted from previously described techniques [13], [29]. Washed radiolabeled cells were suspended in 4 ml of MeOH/100 mM Tris HCl (pH 7.4) containing 4 mM MgCl_2_, 2∶1. Four mls CHCl_3_ were added and the mixture shaken. After centrifugation, the lower CHCl_3_ and upper methanolic phases were recovered. Neutral and negatively charged soluble oligosaccharide material was recovered from the latter phase whereas DLO were recovered from both the former phase and also from the CHCl_3_/MeOH/H_2_O 10∶10∶3 extracts of the interphase proteins. Oligosacharides were released from DLO after mild acid hydrolysis with 0.02N HCl for 30 min at 100°C. The dried upper methanolic phase was taken up in H_2_O and desalted on AG-50 (H^+^ form) and AG-1 (acetate form) ion-exchange columns prior to being loaded onto charcoal columns as previously described. Neutral fOS were eluted from the charcoal with 30% ethanol. Negatively charged material was eluted from the AG-1 resin with 3 M formic acid, and after removing the formic acid under vaccuum, was further treated with either 0.02 N HCl, as described for DLO, or treated with alkaline phosphatase overnight in 100 mM TrisHCl, pH 8.0, at 37°C. Neutralised material was recovered after passage over coupled AG-50/AG-1 resins. Glycoproteins from the 10∶10∶3-extracted protein pellet and the TCA-precipitated glycoproteins recovered from cell culture medium were submitted to protease digestion to yield glycopeptides. Oligosaccharides were released from glycopeptides using endo-β-N-acetylglucosaminidase H from *Streptococcus plicatus* (endoH).

### Analytical procedures

The number of charges associated with oligosaccharide components was evaluated using quaternary aminoethyl (QAE)-Sephadex beads equilibrated in 2 mM Tris base [32]. Material of interest was loaded onto columns in 2 mM Tris base before irrigating the column in the same buffer containing 20, 70, 125, 200, 400 and 1000 mM NaCl. Fractions were collected and assayed for radioactivity by scintillation counting. Neutral fOS and oligosaccharides derived from hydrolysed DLO, negatively charged oligosaccharides and endoH-treated glycopeptides were resolved by thin-layer chromatography (TLC) on silica-coated plastic sheets (0.2 mm thickness) in *n*-propanol/acetic acid/water, 3/3/2 for 16–24h [33]. Radioactive components were detected on X-OMAT AR film by fluorography after spraying the dried TLC plates with En^3^hance and were quantitated by scintillation counting after their elution with water from the silica. After derivatisation with 2-aminopyridine (2-AP) as previously described [29], [34], oligosaccharide mixtures were resolved by HPLC using an amine-bonded silica column (LiChrospher Amino 5 µm, 250 mm ×4.6 mm, Sulpelco Inc). Two eluents were used: eluent A (90% acetonitrile, 10% 30 mM triethylamine, pH 7.3, buffer) and B (10% acetonitrile, 90% 30 mM triethylamine, pH 7.3, buffer). The column was equilibrated in 85% A and 15% B, and after sample injection, was subjected to a linear solvent gradient developed over 80 min until the final solvent mixture of 68% of A and 32% of B was obtained. Radiolabeled oligosaccharides were detected by a Packard 150 TR flow-scintillation analyser. Oligosaccharide-2AP derivatives were also monitored using online fluorimetry and the data generated will be the subject of a separate report.

## Results

### Isolation of negatively charged oligosaccharide-like material from EBV transformed human lymphoblastoid cells and mouse lymphoma cell lines

**Figure 2:**
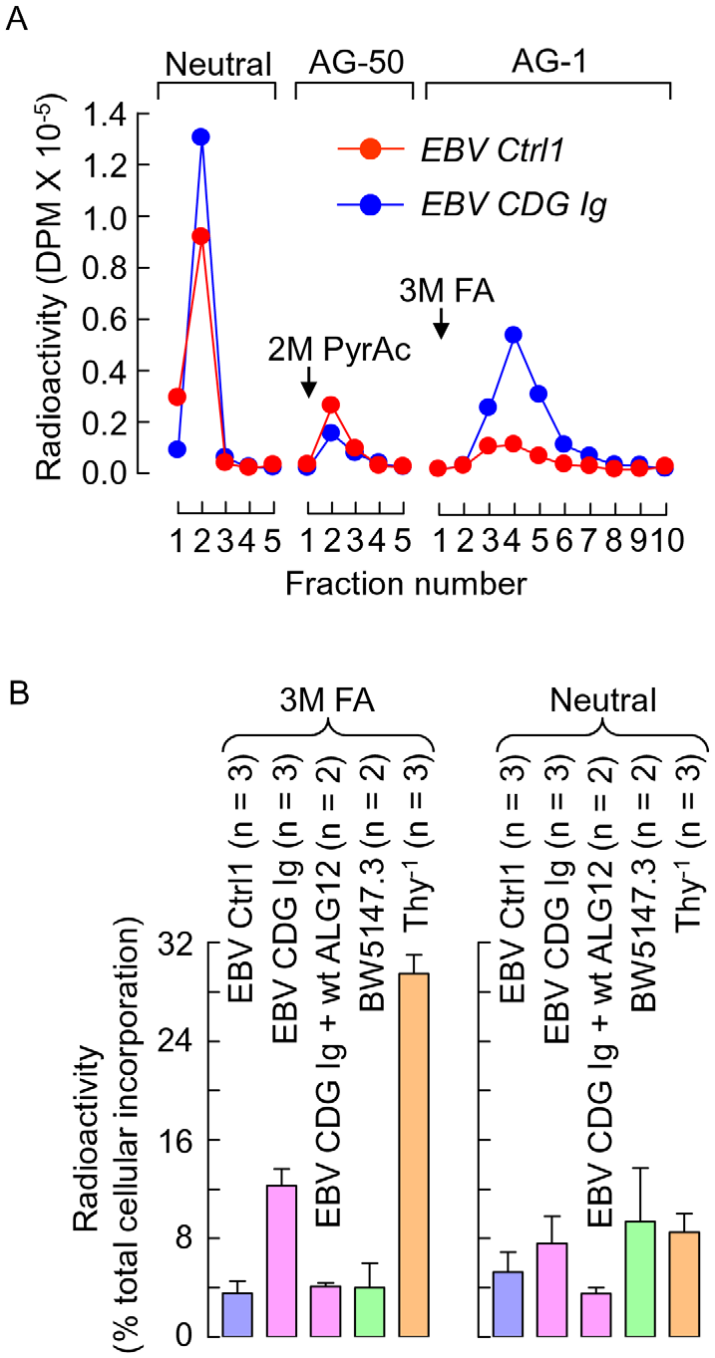
Identification of negatively charged oligosaccharide-like material in EBV-transformed lyphoblasts and murine lymphoma cells. A. EBV lymphoblastoid cells derived from a normal subject (*EBV Ctrl1*) and a patient diagnosed with CDG Ig (ALG12 deficiency, see Fig. 1: *EBV CDG Ig*) were pulse radiolabeled with [2-^3^H]mannose and after extraction with organic solvents as described in Materials and Methods, water soluble components were applied to Biogel P2 columns. Radioactive components, except those eluted in the total inclusion volume (Vi) of the column, were pooled and subjected to ion-exchange chromatography on AG-1(acetate) and AG-50(H^+^) resins. Neutral species passed through both columns (Neutral). Subsequently, the AG-50 column was washed with 2 M pyridine acetate pH, 5.0 (PyrAc) and the AG-1 column was eluted with 3 M formic acid (FA). Fractions were collected and assayed for radioactivity by scintillation counting. B. In addition to the above described cells, EBV CDG Ig cells transduced with wild type ALG12 (EBV CDG Ig + wtALG12) and the parental (BW5147.3) and DPM1-deficient (Thy^-1^) mouse lymphoma cells were radiolabeled as described above. After extraction with organic solvents radioactivity associated with lipid linked oligosaccharides ([^3^H]DLO), glycoproteins ([^3^H]GP) and the oligosaccharide-like materials described above was quantitated by scintillation counting. Radioactivity associated with neutral and FA eluted components is expressed as a percentage of total [2-^3^H]mannose incorporation ([^3^H]DLO + [^3^H]GP + [^3^H]neutral oligosaccharide-like material + [^3^H]FA-eluted oligosaccharide-like material) into the different cell lines.

Neutral free oligosaccharides (fOS) are liberated from either DLO by OST [13], [15] or from glycoproteins by peptide N-glycanase (Ngly1p, [35]) to generate fOSGN2. Negatively charged phosphorylated oligosaccharides (fOSGN2-P) have been identified and are generated from DLO by a DLO pyrophosphatase activity [19]. The release of fOSGN2 [13] and fOSGN2-P [36] from DLO during glycoprotein biosynthesis is thought to occur as a consequence of mechanisms that regulate DLO availability for protein glycosylation [21]. In CDG I, partial blocks in different steps of the DLO biosynthetic pathway lead to accumulations of truncated DLO species [11]. Accordingly, using EBV transformed lymphoblasts derived from either a control subject (EBV ctrl 1 cells) or a patient (EBV CDG Ig cells) with a deficiency in dolichol-P-mannose:Man_7_GlcNAc_2_-PP-dolichol mannosyltransferase (CDG Ig: ALG12-deficiency) we sought evidence for DLO regulation through pyrophosphatase action and the generation of fOSGN2-P. In a first set of experiments, EBV ctrl 1 cells and EBV CDG Ig cells, in which the truncated DLO Man_7_GlcNAc_2_-PP-dolichol is known to accumulate, were pulse radiolabeled with [2-^3^H]mannose and after extraction with organic solvents, water soluble components were submitted to molecular sieve chromatography on Biogel P2. Radioactive material eluting before the inclusion volume of the column was pooled and loaded onto coupled cation and anion exchange chromatography columns as shown in Fig. 2A. Similar amounts of neutral and positively charged components were recovered from the two cell lines. By contrast, a substantially increased amount of negatively charged material was recovered from the anion exchange column after elution with 3M formic acid (FA). After quantitation of this material along with [2-^3^H]DLO, [2-^3^H]glycoproteins and [2-^3^H]fOS, it was noted that when total cellular [2-^3^H]mannose incorporation is considered, [2-^3^H]mannose labelled material in the FA eluates corresponded to ∼4% of total cellular radioactivity in EBV ctrl 1 cells, and this value increased 3 fold in EBV CDG Ig cells (Fig. 2B, left hand panel). Furthermore, after complementing the EBV CDG Ig cells with wild type Alg12p, the quantity of negatively charged [2-^3^H]mannose labelled material was normalised with respect to the EBV ctrl 1 cells. The causal mutations observed in CDG I patients lead to residual activities that enable cells to make low levels of fully mature DLO. In order to evaluate fOSGN2-P generation in lymphoblastoid cells incapable of generating mature DLO, the DPM synthase deficient (null mutation in mouse DPM1 gene; see Fig. 1) mouse lymphoma cell line Thy^-1^ [25], [37] along with its parental cell line (BW5147.3) were examined as described above. As can be seen in the left panel of Fig. 2B, compared to the parental cell line, the mutant cell line generated a 7 fold increase of this negatively charged material. Finally as shown in Fig. 2B (right hand panel) the fluctuations in amounts of neutral fOS between the different cell lines is less pronounced than those noted for the negatively charged components.

### Identification of fOSGN2-P in EBV lymphoblasts and murine lymphoma cells

**Figure 3:**
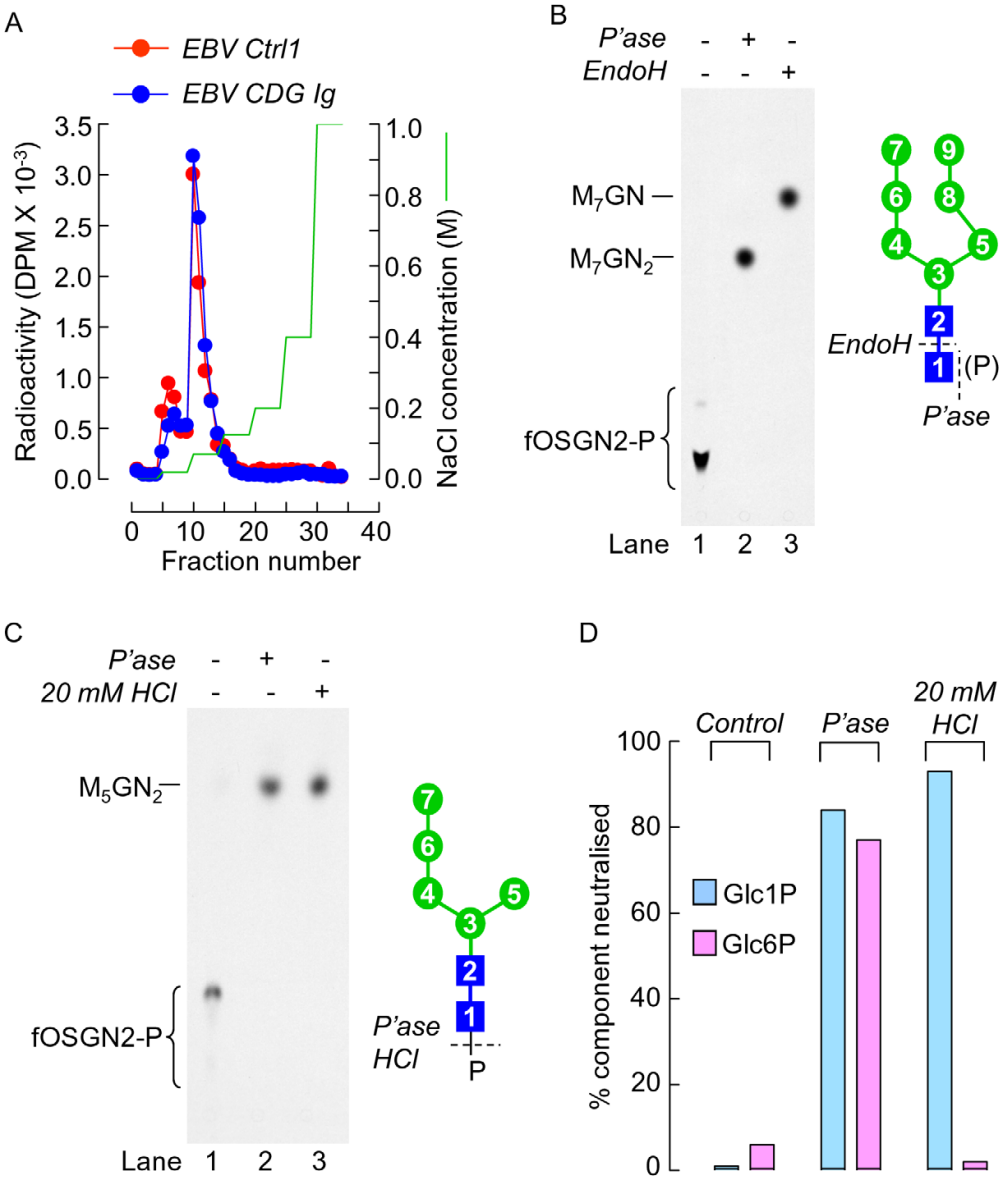
Characterisation of negatively charged oligosaccharide-like material derived from different cell lines. A. Equal amounts of radioactivity associated with material that was eluted from AG-1 columns with 3M FA as described for Fig. 2A were subjected to QAE-Sephadex chromatography as described in Material and Methods. The column was eluted with increasing concentrations of NaCl (indicated on the right hand y axis). Fractions were collected and assayed for radioactivity by scintillation counting. B. Aliquots of the negatively charged oligosaccharide-like material derived from EBV CDG Ig cells were analysed by thin layer chromatography (TLC) before and after treatment with either alkaline phosphatase (*P'ase*) or endo-β-N-acetylglucosaminidase H (*EndoH*). Abbreviations: lines to the left of the TLC fluorograms indicate the migration position of the oligosaccharide (Man_7_GlcNAc_2_; M_7_GN_2_) that was derived by mild acid hydrolysis of Man_7_GlcNAc_2_-PP-dolichol isolated from CDG Ig cells. This oligosaccharide was also treated with endoH to yield Man_7_GlcNAc (M_7_GN). The structure of the oligosaccharide moiety known to occur in the Man_7_GlcNAc_2_-PP-dolichol that accumulates in cells from CDG Ig is shown to the right of the TLC (mannose; green circles, N-acetylglucosamine; blue squares). The di-N-acetylchitobiose moiety of this oligosaccharide is sensitive to endoH. C. Aliquots of the negatively charged oligosaccharide-like material derived from DPM synthase-deficient Thy^-1^ mouse lymphoma cells were analysed by thin layer chromatography (TLC) before and after treatment with either alkaline phosphatase (*P'ase*) or 20 mM HCl. The structure of the oligosaccharide moiety known to occur in the Man_5_GlcNAc_2_-PP-dolichol that accumulates in these cells is shown to the right of the TLC (mannose; green circles, N-acetylglucosamine; blue squares). The di-N-acetylchitobiose moiety of this oligosaccharide is not sensitive to endoH. The line to the left of the fluorograph indicates the migration position of Man_5_GlcNAc_2_ (M_5_GN_2_) that was released by mild acid acid treatment of Man_5_GlcNAc_2_-PP-dolichol derived from Thy^-1^ cells. D. [^14^C]glucose-1-phosphate (Glc1P) and [^14^C]glucose-6-phosphate (Glc6P) were subjected to ion-exchange chromatography on AG-1(acetate) before and after either alkaline phosphatase or mild acid treatment as described in Materials and Methods. Neutralised material was assayed by scintillation counting and expressed as a percentage of input radioactivity.

Next, the nature of the negatively charged radioactive components was examined. Material from each cell line revealed the presence of two peaks of radioactivity after QAE-Sephadex ion-exchange chromatography (Fig. 3A) both of which were neutralised after alkaline phosphatase treatment (results not shown). The minor peak of radioactive material that elutes at 20 mM NaCl was not always present and the origin of this material remains unclear (Durrant-Arico, C. and Moore S.E.H., results not shown). Taking into account that the bulk of the radioactivity was eluted from the column with 70 mM NaCl and was sensitive to alkaline phosphatase, it is concluded that the material contains a single phosphate group [32]. Thin layer chromatography (TLC) of the negatively charged material from the EBV CDG Ig cells (Fig. 3B, lane 1) reveals a predominant slow migrating component and a minor faster migrating species. Both components were neutralised after either alkaline phosphatase or endoH digestion to yield predominantly species that comigrated with Man_7_GlcNAc_2_ and Man_7_GlcNAc species, respectively. These results indicate that the bulk of the negatively charged material derived from EBV CDG Ig cells corresponds to phosphorylated Man_7_GlcNAc_2_ and that the phosphate is attached to the GlcNAc residue at the reducing terminus of the oligosaccharide. Likewise, the negatively charged material derived from the DPM-deficient murine Thy^-1^ lymphoma cells was characterised. This material behaved similarly to the CDG Ig EBV cell derived material upon QAE Sephadex chromatography, was neutralised with alkaline phosphatase to yield predominantly Man_5_GlcNAc_2_ (Fig. 3C, lane 2) but, as expected from the specificity of endoH, was insensitive to this enzyme (results not shown). Furthermore, as the material could be dephosphorylated with dilute HCl (Fig. 3C, lane 3) under conditions where glucose-1-phosphate but not glucose-6-phosphate is dephosphorylated (Fig. 3D), it likely possesses a hemiacetal phosphate rather than a hydroxyl phosphate. Later experiments revealed that the negatively charged material derived from all the cell lines examined was equally sensitive to the alkaline phosphatase and dilute HCl treatments. These results indicate that, compared to control cell lines, the CDG Ig EBV cells and the DPM synthase-deficient mouse lymphoma cells generate increased quantities of the type of phosphorylated oligosaccharides (fOSGN2-P) that have been previously reported to be cleaved from DLO in different cell lines by other groups [16], [17], [18].

### There is a correlation between the quantity of a fOSGN2-P and that of its corresponding DLO only when structures containing 7 or less mannose residues are considered

**Figure 4:**
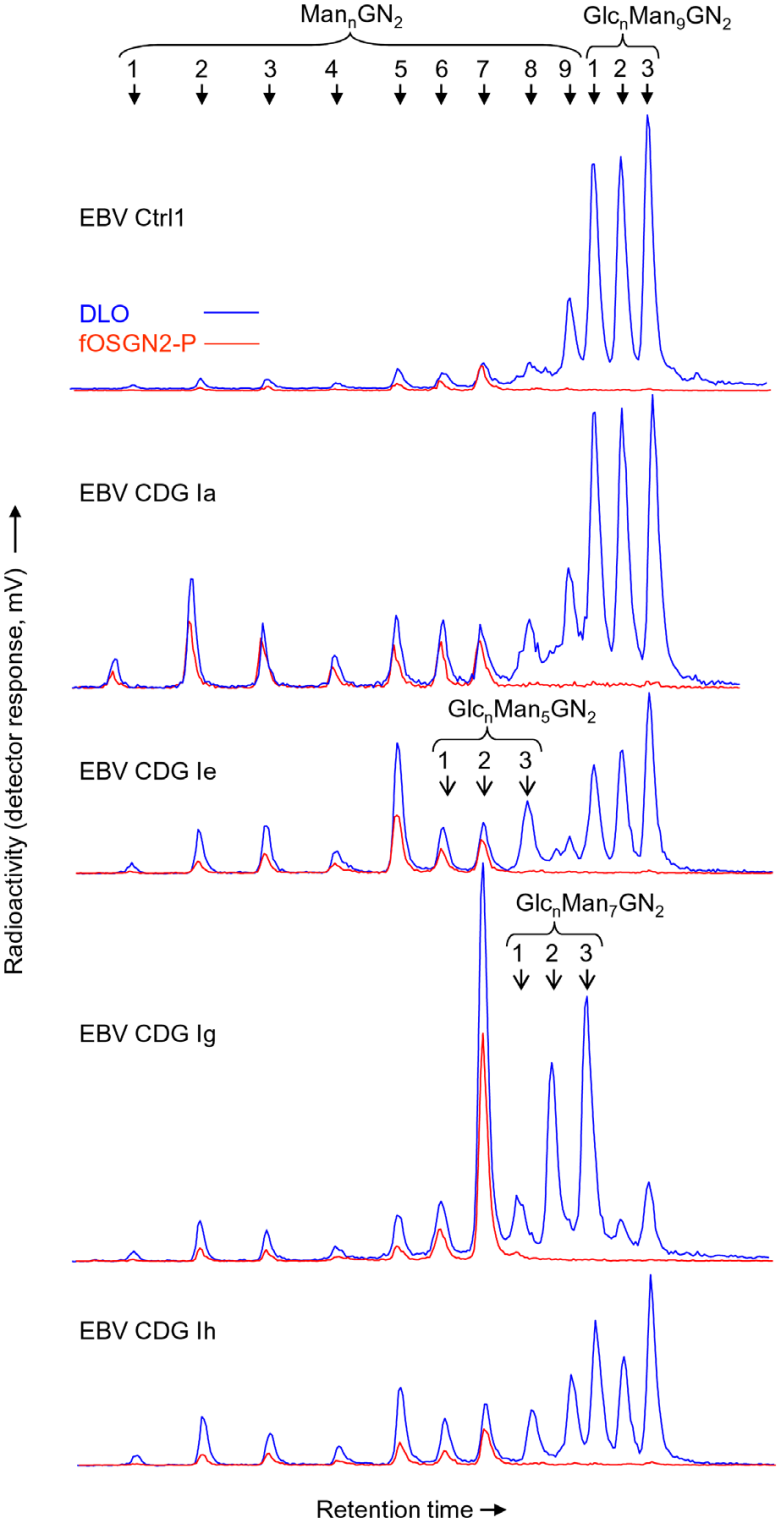
Comparison of oligosaccharide structures generated from DLO and fOSGN2-P isolated from cells of different CDG patients. EBV Ctrl1, EBV CDG Ia, EBV CDG Ie, EBV CDG Ig, and EBV CDG Ih cells were metabolically radiolabelled with [2-^3^H]mannose for 30 min prior to being extracted with organic solvents. DLO and fOSGN2-P were isolated and treated with 20 mM HCl as described in Materials and Methods. Oligosaccharides were subjected to HPLC and resolved components were detected with an on-line flow through scintillation counter. The HPLC traces for DLO- and fOSGN2-P-derived oligosaccharides are blue and red, respectively. The solid arrow heads indicate the elution times of oligosaccharides containing 1–9 residues of mannose (Man_n_GN_2_) and those containing 9 residues of mannose and 1–3 residues of glucose (Glc_n_Man_9_GN_2_). In EBV CDG Ie and Ig cells, glucosylated oligosaccharides containing 5 (Glc_n_Man_5_GN_2_) and 7 (Glc_n_Man_7_GN_2_) residues of mannose, respectively, are also known to occur and their migration positions are indicated with open arrow heads.

In order to examine the origins of fOSGN2-P in more detail, the dephosphorylated structures were compared to the glycone structures of DLO in different control and CDG I EBV cell lines (CDG Ia; PMM2-deficiency, CDG Ig; ALG12-deficiency, CDG Ih; ALG8-deficiency, and CDG Ie; DPM1-deficiency: see Fig. 1) and wild-type and DPM synthase-deficient lymphoma cells. [2-^3^H]Man-labeled fOSGN2-P and DLO were treated with 20 mM HCl and the resulting oligosaccharides were resolved by HPLC and quantitated using on-line flow scintillation analysis as shown in Fig. 4. The two control EBV cell lines and the control mouse lymphoma BW5147 cell line yielded similar profiles (see Fig. 4 upper panel for representative HPLC trace of EBV Ctrl1 cells), and elaborated fOSGN2-P-derived oligosaccharides corresponding to structures containing mainly 2–7 mannose residues (Man_2-7_GlcNAc_2_-P). Although the corresponding species were detected in the DLO pool (Man_2-7_GlcNAc_2_-PP-dolichol), the predominant DLO-deirved species were larger and contained 8–9 residues of mannose and varying numbers of glucose residues (Glc_1-3_Man_8-9_GlcNAc_2_-PP-dolichol). When the chromatographic profiles of DLO-, and fOSGN2-P-derived oligosaccharides obtained from glycosylation deficient cells are inspected (see 4 lower panels in Fig. 4 for representative HPLC traces), similar observations can be made: whatever the origin of the cells, only when structures behaving as oligosaccharides bearing 7 or less residues of mannose are considered is there a correlation between DLO- and fOSGN2-P-derived structures. In addition, it can be seen that where there are accumulations of truncated DLO in the different cell lines (eg. Man_2_GlcNAc_2_-PP-dolichol in CDG Ia cells, Man_5_GlcNAc_2_-PP-dolichol in CDG Ie cells and Man_7_GlcNAc_2_-PP-dolichol in CDG Ig cells) there are increases in the corresponding fOSGN2-P species. In EBV CDG Ie cells, fOSGN2-P derived structures that co-elute with DLO derived Glc_1-2_Man_5_GlcNAc_2_-P were identified. However, the mutation in the DPM1 gene in these cells is leaky, leading to residual synthesis of fully mannosylated DLO. Accordingly, DLO and fOSGN2-P derived components that elute as Glc_1-2_Man_5_GlcNAc_2_-P are potentially mixtures containing also Man_6-7_GlcNAc_2_ species. By contrast to EBV CDG Ie cells, in the Thy^-1^ cells, DPM synthase is inactive leading to an absence of DLO structures containing more than 5 mannose residues. In these cells a fOSGN2-P derived structure comigrating with DLO-derived Glc_1_Man_5_GlcNAc_2_ was detected, but although substantial quantities of DLO-derived Glc_2-3_Man_5_GlcNAc_2_ were noted corresponding structures derived from fOSGN2-P occured at very low levels (Peric, D. and Moore, S. unpublished results). Accordingly, despite the abundance of glucosylated DLO in all cell lines tested, glucosylated fOSGN2-P, if present, are under represented in the total fOSGN2-P pool.

**Figure 5:**
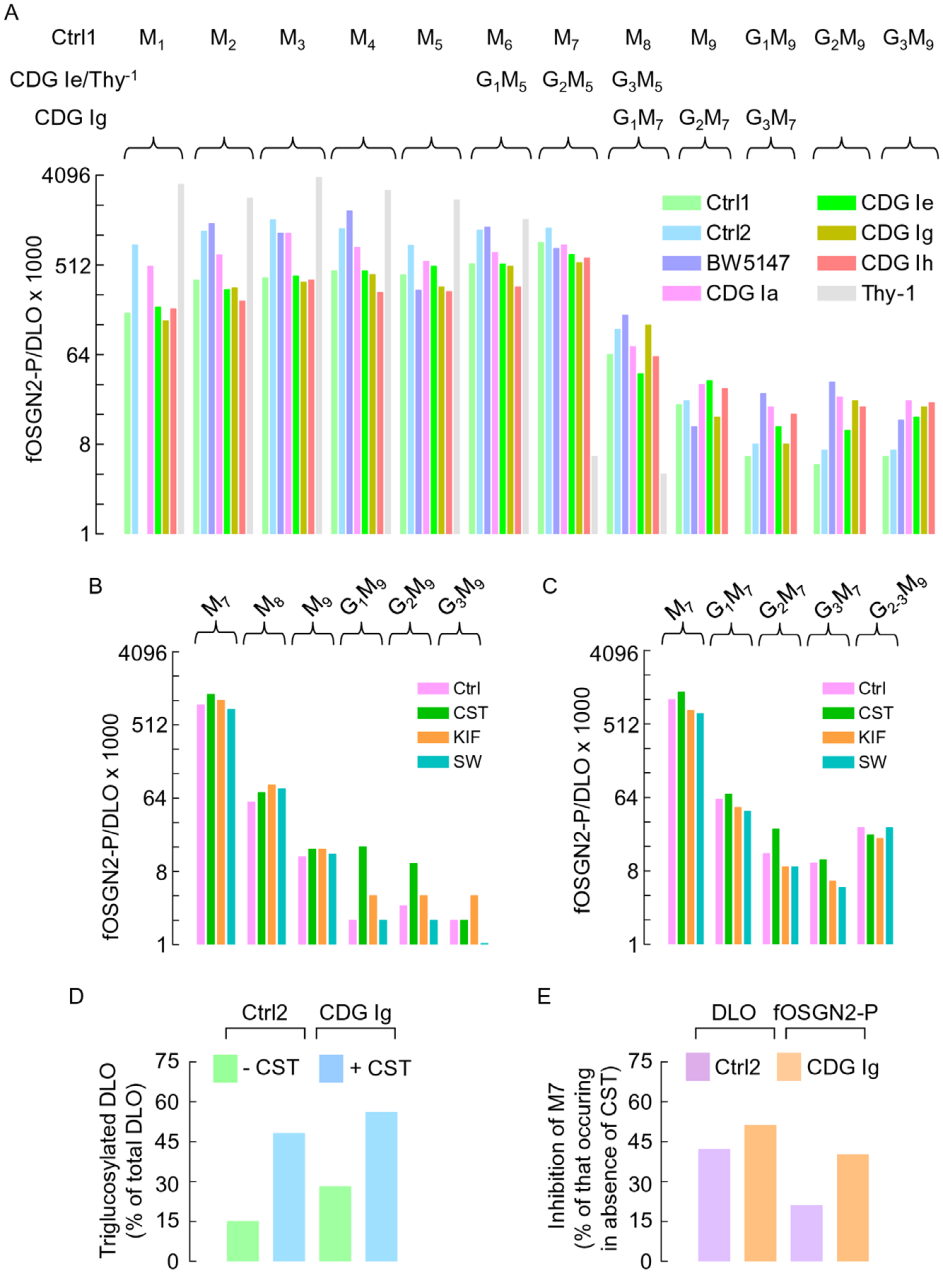
Computation of the ratio of fOSGN-P to DLO for different oligosaccharide structures observed in the different cell lines cultivated in the absence or presence of glucosidase and mannosidase inhibitors. A. Oligosaccharides derived from DLO and fOSG2-P isolated from EBV Ctrl1, EBV Ctrl2, EBV CDG Ia, EBV CDG Ie, EBV CDG Ig, EBV CDG Ih, BW5417.3 and Thy^-1^ cells were prepared and resolved by HPLC as described in Fig. 4. Peak areas were recorded and used to derive the ratio fOSGN2-P/DLO for each oligosaccharide structure. These values were multiplied by 1000 and imposed on a logarithmic scale. Abbreviations: M_1-9_; Man_1-9_GlcNAc_2_, G_1-3_M_1-9_; Glc_1-3_Man_1-9_GlcNAc_2_, G_1-3_M_1-7_; Glc_1-3_Man_1-7_GlcNAc_2_, G_1-3_M_1-5_; Glc_1-3_Man_1-5_GlcNAc_2_. In a separate experiment EBV Ctrl2 (B) and EBV CDG Ig (C) cells were preincubated and then radiolabeled as described above, in the presence of the mannosidase inhibitors swainsonine (SW) and kifunensin (KIF) or the glucosidase inhibitor castanospermine (CST). Oligosaccharides derived from DLO and fOSGN2-P were resolved by TLC and, after elution of radioactive components from the chromatography plates followed by scintillation counting, the ratio fOSGN2-P/DLO for the oligosaccharide structures M_7_–G_3_M_9_ were generated and presented as described above. D. Using data from the experiment described in B and C the percentage of total DLO species occurring as triglucosylated species was computed for EBV Ctrl2 and EBV CDG Ig cells radiolabeled in either the absence or presence of CST. E. Using data from the experiment described in B and C, DLO-, and fOSGN2-P-derived oligosaccharides possessing 7 residues of mannose were quantitated. The amounts of these components that were generated in cells treated with CST have been expressed as a percentage of those generated in cells radiolabeled in the absence of this reagent.

The ensemble of these data is consolidated as shown in Fig. 5A where for each oligosaccharide structure the ratio of the quantities of the corresponding fOSGN2-P and DLO has been computed and multiplied by 1000. It can be seen that this value is lowest for Glc_1-3_Man_9_GlcNAc_2_-P/Glc_1-3_Man_9_GlcNAc_2_-PP-dolichol, begins to rise with Man_8-9_GlcNAc_2_-P/Man_8-9_GlcNAc_2_-PP-dolichol and is maximal with Man_7_GlcNAc_2_-P/Man_7_GlcNAc_2_-PP-dolichol. Accordingly, the structures and quantities of fOS-P are related to the structures and quantities of DLO providing that the latter components possess seven or less mannose residues.

When EBV Ctrl2 cells and EBV CDG Ig cells were metabolically radiolabeled in the presence of the class I and II mannosidase inhibitors KIF and SW, respectively, the ratios of the corresponding DLO and fOSGN2-P species were not strikingly affected, arguing against the possibility of rapid demannosylation of more fully mannosylated fOSGN2-P (Fig. 5B and C). Furthermore, neither inhibitor affected the quantity of either the DLO or fOSGN2-P species that were generated (Moore, S., results not shown). By contrast, the ER glucosidase I and II inhibitor, castanospermine (CST), provoked 3 and 2 fold increases in the proportion of DLO species that are triglucosylated in EBV Ctrl2 cells and EBV CDG Ig cells, respectively (Fig. 5D). These changes were accompanied by 42% and 21% inhibitions of Man_7_GlcNAc_2_-PP-dolichol and Man_7_GlcNAc_2_-P, respectively in EBV Ctrl2 cells, and 51% and 40% inhibitions of Man_7_GlcNAc_2_-PP-dolichol and Man_7_GlcNAc_2_-P, respectively in EBV CDG Ig cells (Fig. 5E). Finally, in EBV Ctrl2 cells (Fig. 5B), CST did increase the Glc_1-2_Man_9_GlcNAc_2_-P/Glc_1-2_Man_9_GlcNAc_2_-PP-dolichol ratios, but these elevated ratios were still between one and two orders of magnitude less than that observed for Man_7_GlcNAc_2_-P/Man_7_GlcNAc_2_-PP-dolichol. These results confirm a previous report demonstrating that glucosidase inhibition did not lead to the appearance of glucosylated fOSGN2-P, but did reduce formation of non-glucosylated fOSGN2-P [20].

### Man_7_GlcNAc_2_-P occurs predominantly in the cytosolic fraction of EBV-transformed lymphoblast cells derived from a patient with ALG12 deficiency

**Figure 6:**
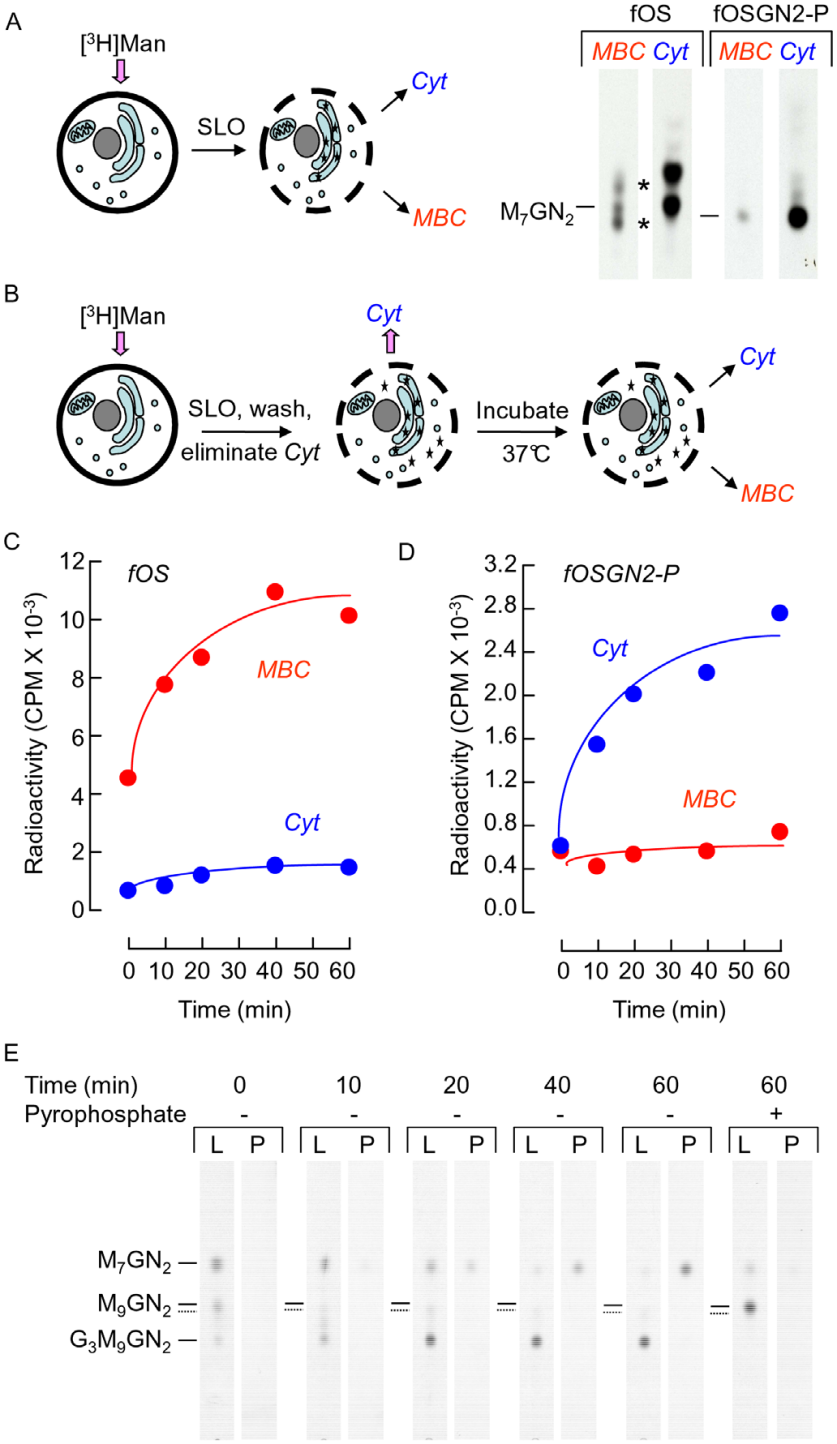
fOSGN2-P are generated in streptolysin O-permeabilised cells. A. EBV CDG Ig cells were pulse radiolabeled for 30 min with [2-^3^H]mannose and then permeabilised with streptolysin O (SLO) at 4°C in permeabilisation buffer as described in Materials and Methods. After centrifugation fOSGN2-P and neutral fOS were recovered from both the supernatant containing cytosolic components (*Cyt*) and the permeabilised cell pellet containing intact membrane bound compartments (*MBC*). After dephosphorylation with mild acid treatment fOSGN2-P and fOS were examined by TLC. The migration position of Man_7_GlcNAc_2_ (M_7_GN_2_), derived by mild acid hydrolysis of Man_7_GlcNAc_2_-PP-dolichol isolated from CDG Ig cells, is indicated to the left of each pair of chromatograms. B. EBV CDG Ig cells were pulse radiolabeled for 30 min with [2-^3^H]mannose and then permeabilised with SLO in incubation buffer as described in Materials and Methods. After incubation of permeabilised cells in the presence of 20 µM each of UDP-Glc, GDP-Man, and UDP-GlcNAc for various times at 37°C, *Cyt* and *MBC* fractions were generated as described above. Neutral fOS (C) and fOSGN2-P (D) were recovered from the *Cyt* and *MBC* fractions and assayed by scintillation counting. E. DLO (L) and fOSGN2-P (P) recovered from the incubations described in C and D were hydrolysed using mild acid treatment and the resulting oligosaccharides were analysed by TLC. Pyrophosphate 10 mM was added to the indicated reaction mixture. The migration positions of standard oligosaccharides are indicated by the solid lines to the left of the chromatograms. The oligosaccharide migrating slightly slower than Man_9_GlcNAc_2_ (indicated with the dotted line) was not characterised but migrates as Glc_1_Man_9_GlcNAc_2_ or Glc_3_Man_7_GlcNAc_2_. The TLC plate on which DLO-derived oligosaccharides were resolved was exposed to film for 7 days whereas that on which fOSGN2-P-derived oligosaccharides were resolved was exposed for 14 days.

In order to address the mechanism underlying fOSGN2-P generation, the subcellular localisation of Man_7_GlcNAc_2_-P was explored in EBV CDG Ig cells. After metabolic radiolabeling for 30 min cells were permeabilised with streptolysin O (SLO) on ice. The procedure employed is known to specifically permeabilise the plasma membrane of cells [29], [30], [38], and after centrifugation, the supernatant contains soluble cytosolic components and the pellet comprises permeabilised cells possessing intact intracellular membrane bound compartments (MBC). Data presented in Fig. 6A demonstrate that both neutral fOSGN2-P and fOS are predominantly recovered from the cytosolic compartment. However, inspection of the neutral fOS recovered from MBC revealed the presence of two fOS species (marked with asterisks) that were not observed in the cytosol fraction, attesting to selectivity of the SLO permeabilisation process.

### An *in vitro* assay to monitor Man_7_GlcNAc_2_-P generation using SLO-permeabilised CDG Ig cells

In order to further investigate the compartmentalisation of fOSGN2-P generation an *in vitro* assay was established for the generation of Man_7_GlcNAc_2_-P using SLO-permeabilised EBV-CDG Ig cells. It has been demonstrated that after specific permeabilisation of the plasma membrane of various cell lines with SLO, vesicle-mediated intracellular transport of proteins [39], protein N-glycosylation [31], [40] and non-vesicular ER-to-cytosol transport of neutral fOS [31] do not occur unless cytosolic factors are added back to the permeabilised cells. Aspects of the dolichol cycle have also been studied in SLO permeabilised EBV cells from control subjects and CDG patients and it was shown that DLO glucosylation is maintained when UDP-Glc is added to the incubation mixtures [22]. Accordingly, in order to reproduce observations made in intact cells, permeabilised EBV-CDG Ig cells were incubated for up to 1 h with UDP-Glc, GDP-Man and UDP-GlcNAc using the protocol outlined in Fig. 6B. As indicated in Fig. 6C and D, both neutral fOS and fOSGN2-P are generated in a time dependent manner, but whereas the former are generated predominantly in the MBC fraction, the latter appear predominantly in the cytosolic fraction. TLC analysis of the DLO-, and fOSGN2-P-derived oligosaccharides generated during the incubations is shown in Fig. 6E. Within 10 min DLO intermediates possessing 9 residues of mannose are predominantly triglucosylated, and after 60 min Glc_3_Man_9_GlcNAc_2_-PP-dol is the major DLO with smaller amounts of Glc_3_Man_7_GlcNAc_2_-PP-dol and Man_7_GlcNAc_2_-PP-dol also being present. Despite the presence of fully glucosylated DLO species, Man_7_GlcNAc_2_-P was the only fOSGN2-P detected. It has been reported that the DLO pyrophosphatase activity is inhibited by pyrophosphate [19]. As demonstrated in Fig. 6E, this reagent does reduce Man_7_GlcNAc_2_-P generation but also leads to the accumulation of a DLO intermediate behaving as Man_9_GlcNAc_2_-PP-dolichol. Indeed, after jack bean α-mannosidase digestion of radioactive components eluted from this region of the chromatogram, it was ascertained that the predominant component was not glucosylated (Durrant-Arico, C. and Moore, S. results not shown). These data suggest that in addition to inhibiting the DLO pyrophosphatase, pyrophosphate blocks DLO glucosylation. The appearance of fully mannosylated DLO intermediates in cells from this CDG Ig patient is not unexpected because the mutation in the ALG12-encoded mannosyltransferase is leaky [28]. In the context of the *in vitro* system reported here where DLO utilisation is strikingly reduced, Man_7_GlcNAc_2_-PP-dolichol elongation by the defective ALG12-encoded mannosyltransferase may be significantly enhanced. To summarise, Man_7_GlcNAc_2_-P is generated in SLO-permeabilised CDG Ig cells and the selectivity of fOSGN2-P generation reproduces that observed in intact cells.

### N-glycosylation and fOSGN2-P generation compete for the same DLO pool

**Figure 7:**
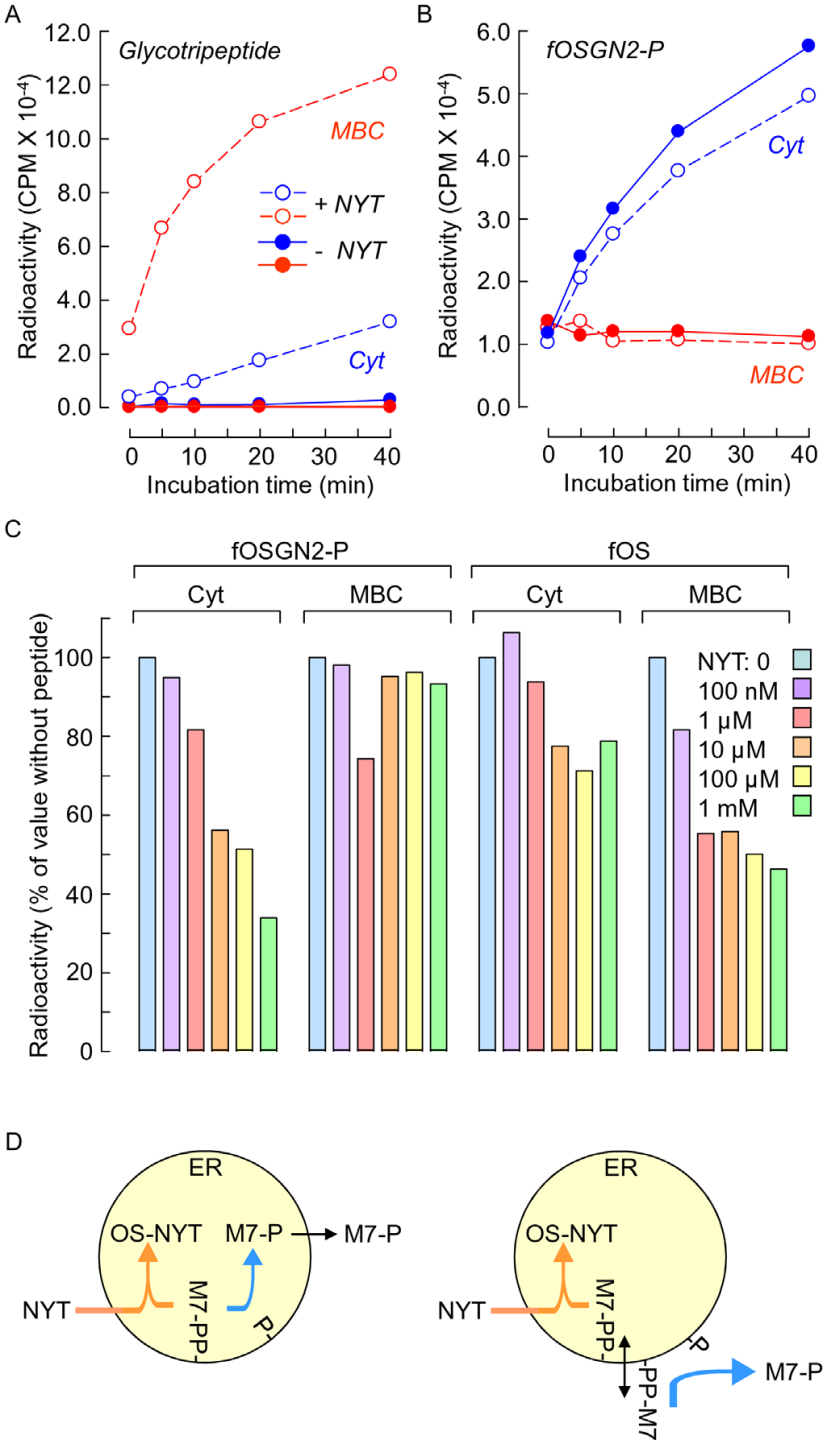
A tripeptide containing the N-glycosylation concensus sequence inhibits fOSGN2-P generation in permeabilised cell incubations. Permeabilised EBV CDG Ig cells were prepared as described for Fig. 6B and incubated in the absence (*−NYT*) or presence of 1 µM Ac-Asn-Tyr-Thr-NH_2_ (*+ NYT*) for 60 min. The resulting [2-^3^H]mannose-labelled glycotripeptide (A) and fOSGN2-P (B) were isolated from both the MBC (*MBC*) and cytosolic (*Cyt*) fractions as described in Materials and Methods and assayed by scintillation counting. C. In a different experiment permeabilised cells were incubated in the absence (NYT: 0) or the indicated concentrations of the tripeptide before isolation and quantitation of MBC- or cytosol-situated neutral fOS (fOS) and fOSGN2-P. The quantity of the two components recovered from each cellular compartment is expressed as a percentage of that occurring in the absence of tripeptide. D. Data shown in Figs. 6 and 7 indicate that Man_7_GlcNAc_2_-P is either generated in the lumen of the ER and then transported into the cytosolic compartment by a highly efficient process (left panel), or is liberated on the cytosolic face of the ER. Although Man_7_GlcNAc_2_-PP-dol is thought to be synthesised on the luminal face of the ER, *in vitro* experiments suggest that this structure can be potentially flipped onto the cytosolic face of the ER (right panel).

Although DLO pyrophosphatase activity has been identified in various microsome preparations [13], [19] we wanted to demonstrate that Man_7_GlcNAc_2_-P generation and ER-mediated polypeptide N-glycosylation [41] occur at the same subcellular localisation in permeabilised cells. Accordingly, if the DLO pool required for polypeptide N-glycosylation is functionally linked to that which gives rise to fOSGN2-P, then addition of a tripeptide (Ac-Asn-Tyr-Thr-NH_2_, NYT) containing the N-glycosylation concensus sequence to the vesicular transport-incompetent permeabilised cells could potentially deplete the DLO pool giving rise to fOSGN2-P and therefore inhibit fOSGN2-P generation. As indicated in Fig. 7A, when permeabilised cells are incubated with 1 µM NYT, there is a rapid generation of glycosylated NYT in the MBC accompanied by the appearance of smaller quantities of this component in the cytosol, indicating, as expected, that under these assay conditions peptide N-glycosylation occurs, and the ER membrane represents a significant barrier for the movement of the resulting glycopeptide into the cytosolic compartment [31]. In the same incubations the quantity of fOSGN2-P only increased in the cytosolic compartment, and this increase was reduced by ∼20% when the tripeptide was present (Fig. 7B). Finally, as shown in Fig. 7C, the concentration dependence of the inhibition of fOSGN2-P by tripeptide was evaluated and compared to that of the inhibition of neutral fOS in the same incubations. The appearance of cytosolic fOSGN2-P was inhibited in a dose dependent manner by NYT, but the quantity of MBC-associated fOSGN2-P remains quite stable even at high tripeptide concentrations. A proportion of neutral fOS are thought to be generated by OST when glycosylation acceptor polypeptides are limiting [13], [15]. In accordance with this, addition of NYT also causes a dose-dependent inhibition of neutral fOS in both the MBC and cytosolic compartments (Fig. 7C). The ensemble of these results indicate that, in SLO permeabilised EBV CDG Ig cells, Man_7_GlcNAc_2_-P generation is an ER-associated event and that this structure is either generated in the lumen followed by highly efficient ER-to-cytosol transport (Fig. 7D, left panel), or is cleaved from cytosolically disposed Man_7_GlcNAc_2_-PP-dolichol (Fig. 7D, right panel).

## Discussion

fOSGN2-P were first identified in microsomes derived from mouse myeloma tumour MOPC-46B cells incubated with dolichol-P-[^14^C]Man [18]. These incubations yielded [^14^C]Man_5_GlcNAc_2_-PP-dolichol, [^14^C]Man_5_GlcNAc_2_-P and [^14^C]glycoproteins, and time course studies led to the conclusion that, whereas the DLO was the precursor for protein glycosylation, the fOSGN2-P was a degradation product of the DLO [18]. In intact rat spleen lymphocytes, Man_9-8_GlcNAc_2_-P were the major fOSGN2-P identified despite the presence of substantial quantities of glucosylated DLO [20]. Inhibition of glucosidase activities did not unmask the presence of glucosylated fOSGN2-P but did reduce the appearance of non glucosylated fOSGN2-P [20]. In DPM synthase deficient CHO cells in which glucosylated DLO intermediates predominate over their non-glucosylated counterparts, only Man_5_GlcNAc_2_-P and Man_2_GlcNAc_2_-P were identified [21]. Here we demonstrate the presence of fOSGN2-P in EBV lymphoblastoid and mouse lymphoma cells. We have also detected fOSGN2-P in skin biopsy fibroblasts from control subjects and CDG I patients. However, due to variable amounts of truncated DLO in cells from normal subjects, interpretation of data from these cells is difficult. Under our cell culture and metabolic radiolabeling conditions it was found that the EBV transformed lymphoblasts gave more reproducible results. In EBV cells from control subjects and parental mouse lymphoma cells Man_1-7_GlcNAc_2_-P predominated despite the fact that the major DLO species were found to be Glc_0-3_Man_9_GlcNAc_2_-PP-dolichol. In cells from CDG I patients the same fOSGN2-P species were observed but they occured at higher levels which appeared to reflect higher levels of truncated DLO intermediates in these cells. Accordingly, the increased fOSGN2-P generation that occurs in CDG cells appears to result from an elevated flux of substrate through a pathway already operating at a low level in normal cells. It was found that CST increased the proportion of triglucosylated Man_9_GlcNAc_2_-PP-dolichol in control cells and triglucosylated Man_7_GlcNAc_2_-PP-dolichol in CDG Ig cells, and in both cases reduced the amount of Man_7_GlcNAc_2_-P. Accordingly, it can be concluded that the glucosylation of some immature DLO intermediates protects them from giving rise to fOSGN2-P. Because CST does not cause complete inhibition of fOSGN2-P generation, the previously described DLO glucosylation/deglucosylation cycle [14] that is known to occur in EBV cells [22] is not a feature of all fOSGN2-P generation. Although our results demonstrating the paucity of glucosylated fOSGN2-P, even under conditions of glucosidase inhibition, are in agreement with other studies [20], [21], the cut-off structure for efficient fOSGN2-P generation from DLO in our studies appears to be Man_7_GlcNAc_2_-PP-dolichol rather than the Man_9-8_GlcNAc_2_-PP-dolichol structures noted by others [19], [20], [36]. By contrast to the glucosidase inhibitor, the class I and II mannosidase inhibitors, kifunensin and swainsonine had no effect on either DLO biosynthesis or fOSGN2-P generation, suggesting that, unless an unusual mannosidase activity is involved, Man_7_GlcNAc_2_-P generation does not involve demannosylation of more fully mannosylated DLO or fOSGN2-P. Under normal circumstances it is thought that DLO biosynthesis follows the pathway shown in Fig. 1 but it may be more complex. In analogous fashion to the processing of N-glycans during glycoprotein folding, DLO intermediates may be subjected to different processes depending on their residence time in the lumen of the ER. For example, under normal circumstances Man_7_GlcNAc_2_-PP-dolichol is rapidly converted to Man_8_GlcNAc_2_-PP-dolichol by the DPM-requiring Alg12p mannosyltransferase [4]. If this reaction is slowed down, Man_7_GlcNAc_2_-PP-dolichol could be involved in several less efficient reactions such as glucosylation by DPG-requiring Alg6p glucosyltransferase, OST-mediated transfer of Man_7_GlcNAc_2_ onto polypeptide or water to yield either glycopeptides or neutral fOS, respectively, and finally, *in vitro* evidence suggests that ER luminal Man_7_GlcNAc_2_-PP-dolichol may be able to slowly flip back onto the cytosolic face of the ER [8]. Our results indicate that Man_7_GlcNAc_2_-PP-dolichol can also give rise to Man_7_GlcNAc_2_-P. Clearly, the eventual fate of the Man_7_GlcNAc_2_-PP-dolichol will depend on the relative rates of these competing reactions. The question of why immature DLO intermediates are capable of giving rise to fOSGN2-P rather than fully mature DLO in normal cells remains to be elucidated, but in CDG I cells two factors may come into play. First, defects in enzymic steps of the DLO pathway will clearly favour consumption of the accumulated DLO intermediate by the type of less efficient secondary reactions described above. Second, in CDG I cells a more generalised perturbation of ER function, caused by, for example, misfolded glycoproteins, may lead to a slow down in certain steps of the DLO cycle. To conclude, our results show that in EBV lymphoblastoid cells from normal subjects and mouse lymphoma cells fOSGN2-P generation occurs at low levels, but in EBV CDG I cells and DPM1-deficient mouse lympoma cells increases in immature DLO intermediates lead to increases in fOSGN2-P generation.

### The selectivity and subcellular localisation of fOSGN2-P generation

Three hypotheses could explain the selectivity of fOSGN2-P generation that we observe in EBV lymphoblastoid or mouse lymphoma cells. First, the pyrophosphatase activity may show specificity towards non-glucosylated, hypomannosylated, DLO intermediates. However, it has been demonstrated that calf thyroid microsomes are capable of yielding fOSGN2-P from exogenous Glc_3_Man_9_GlcNAc_2_-PP-dolichol and a yeast microsomal pyrophosphatase activity is capable of generating Man_8_GlcNAc_2_-P from exogenous Man_8_GlcNAc_2_-PP-dolichol. Second, all DLO structures could yield corresponding fOSGN2-P structures but either a phosphatase or cytosolic Engase1p could potentially specifically neutralise Glc_3-0_Man_9-8_GlcNAc_2_-P structures to yield the corresponding fOSGN2 or fOSGN, respectively, which would go undetected in our assays. This hypothesis can not be excluded but as human cytosolic Engase1p can cleave triglucosylated fOSGN2 (Chantret, I. and Moore, S., manuscript in preparation) the presence of a highly selective fOSGN2-P would have to be proposed. Third, the pyrophosphatase activity may be compartmentalised differently to more fully mature DLO intermediates, and the selectivity of the putative mechanism that regulates this DLO compartmentalisation would underly the selectivity of fOSGN-P generation. This hypothesis is difficult to evaluate because the subcellular localisation of the pyrophosphatase is not clear. Although the subcellular site for the generation of Man_9-8_GlcNAc_2_-P has not been investigated, the lumenal orientation Man_9-8_GlcNAc_2_-PP-dolichol has led to the assumption of an ER luminal pyrophosphatase activity [19]. On the other hand Man_5_GlcNAc_2_-P and Man_2_GlcNAc_2_-P were only recovered in the cytosol fraction of DPM-deficient CHO cells whose plasma membrane is permeabilised. As DLO intermediates containing 5 or less mannose residues are generated on the cytosolic face of the ER, the pyrophosphatase activity was proposed to work at the cytosolic face of this organelle [42].

### Is Man_7_GlcNAc_2_-P generated within the ER or in the cytoplasm or in both compartments?

Using an *in vitro* assay we show that fOSGN2-P generation is reduced when permeabilised cells are incubated with a peptide containing the N-glycosylation consensus sequence. This result demonstrates that the DLO pool that gives rise to fOSGN2-P and the pool which is required for peptide glycosylation are functionally related. Furthermore, as peptide N-glycosylation is mediated by OST in the ER and vesicular transport is not supported in SLO permeabilised cells, these data indicate that fOSGN2-P generation is a property of the ER itself or of some contiguous membrane structure. When cells are metabolically radiolabeled and then permeabilised with SLO at 4°C we noted that although ∼80% of Man_7_GlcNAc_2_-P was recovered in the cytosol fraction, trypan blue exclusion studies indicated that greater than 95% of cells had been permeabilised. Three hypotheses may be postulated to explain these data. First, under our permeabilisation conditions, Man_7_GlcNAc_2_-P may be less permeant than trypan blue. Second, a Man_7_GlcNAc_2_-P pool could be generated within an MBC. Third, cytosolic Man_7_GlcNAc_2_-P could bind to exposed sites of the permeabilised cells. Whatever the explanation behind the localisation of MBC-associated Man_7_GlcNAc_2_-P, *in vitro* incubations reveal that this fOSGN2-P pool is stable and little affected by the presence of the glycosylation acceptor peptide. By contrast, the amount of cytosolic Man_7_GlcNAc_2_-P increases 6 fold during such incubations and this production is sensitive to the presence of the glycosylation acceptor peptide. Accordingly, we were unable to detect the precursor/product relationship between MBC-, and cytosol-situated Man_7_GlcNAc_2_-P that would be expected from ER-to-cytosol transport of this structure. Both glycopeptides [43], [44], [45], [46] and fOS [31], [47] have been reported to be transported out of the ER into the cytosol, but these processes require cytosolic factors such as GTP and ATP and, as expected, in our *in vitro* assay for the generation of Man_7_GlcNAc_2_-P which is carried out in the absence of such molecules, a glycotripeptide and fOS remain predominantly within the MBC.

### Potential mechanisms for the appearance of cytosolic Man_7_GlcNAc_2_-P

What mechanism could account for the generation of cytosolic Man_7_GlcNAc_2_-P from luminal Man_7_GlcNAc_2_-PP-dolichol without the appearance of luminal Man_7_GlcNAc_2_-P under conditions where other known transport processes, if present, operate so inefficiently that their substrates accumulate in the ER? First, a luminal pyrophosphatase activity could be tightly coupled to an ER-to-cytosol transport process allowing efficient molecular channelling of the pyrophosphatase product to the transporter resulting in an undetectable pool of luminal Man_7_GlcNAc_2_-P (Fig. 7D, left panel). Second, a flippase could retrotranslocate Man_7_GlcNAc_2_-PP-dolichol from the luminal to the cytosolic face of the ER thereby exposing the DLO intermediate to a pyrophosphatase whose active site is cytosolic (Fig. 7D, right panel). Indeed, there is evidence for ATP-independent, protein-mediated, flipping of Man_7_GlcNAc_2_-PP-dolichol across artificial sealed liposomes, although in these studies, Man_5_GlcNAc_2_-PP-dolichol appeared to be the best substrate for this activity [8]. Thus, if it is hypothesesied that the DLO pyrophosphatase acts at the cytosolic face of the ER, flippase-mediated distribution of DLO intermediates across the ER membrane could conceivably contribute to the apparent selectivity of fOSGN2-P generation.

To conclude, fOSGN2-P have been observed in EBV lymphoblastoid cells from control subjects and CDG I patients and murine lymphoma cells. In cells with glycosylation deficits where non-glucosylated DLO intermediates containing 7 or less mannose residues accumulate, increased fOSGN2-P generation is observed. The functional link between DLO pools required for N-glycosylation and fOSGN2-P generation in permeabilised cells indicates that they are contiguous and substantiates the hypothesis that pyrophosphatase-mediated cleavage of DLO intermediates could yield rapidly recyclable dolichol-P. The mechanisms underlying fOSGN2-P generation appear complex and reveal a novel ER-to-cytosol translocation process for either fOSGN2-P or DLO.
